# Analysis of Structures and Epitopes of Surface Antigen Glycoproteins Expressed in Bradyzoites of *Toxoplasma gondii*

**DOI:** 10.1155/2013/165342

**Published:** 2013-03-21

**Authors:** Hua Cong, Min Zhang, Qingli Zhang, Jing Gong, Haizi Cong, Qing Xin, Shenyi He

**Affiliations:** ^1^Department of Human Parasitology, School of Medicine, Shandong University, No. 44 Wenhuaxi Road, Jinan, Shandong 250012, China; ^2^Laboratory of Morphology, School of Medicine, Shandong University, No. 44 Wenhuaxi Road, Jinan, Shandong 250012, China; ^3^Cancer Research Center, School of Medicine, Shandong University, No. 44 Wenhuaxi Road, Jinan, Shandong 250012, China; ^4^Department of College English, Shandong University, No. 44 Wenhuaxi Road, Jinan, Shandong 250012, China; ^5^School Hospital of Shandong University, No. 73 Jingshi Road, Jinan, Shandong 250012, China

## Abstract

*Toxoplasma gondii* is a protozoan parasite capable of infecting humans and animals. Surface antigen glycoproteins, SAG2C, -2D, -2X, and -2Y, are expressed on the surface of bradyzoites. These antigens have been shown to protect bradyzoites against immune responses during chronic infections. We studied structures of SAG2C, -2D, -2X, and -2Y proteins using bioinformatics methods. The protein sequence alignment was performed by T-Coffee method. Secondary structural and functional domains were predicted using software PSIPRED v3.0 and SMART software, and 3D models of proteins were constructed and compared using the I-TASSER server, VMD, and SWISS-spdbv. Our results showed that SAG2C, -2D, -2X, and -2Y are highly homologous proteins. They share the same conserved peptides and HLA-I restricted epitopes. The similarity in structure and domains indicated putative common functions that might stimulate similar immune response in hosts. The conserved peptides and HLA-restricted epitopes could provide important insights on vaccine study and the diagnosis of this disease.

## 1. Introduction

*Toxoplasma gondii* (*T. gondii*) is a species of parasitic protozoa in the genus *Toxoplasma* that can be carried by many warm-blooded animals including humans [1]. There are three infectious stages in a complex life cycle of *T. gondii*: the tachyzoites, the bradyzoites, and the sporozoites [2]. A bradyzoite is a slowly replicating version of the parasite, which is responsible for chronic infection of *T. gondii* [3]. In chronic toxoplasmosis, the parasitophorous vacuoles containing the reproductive bradyzoites form cysts in the tissues of the muscles and brain [4].

The surface antigen of *T. gondii* that plays roles in the processes of host cell attachment and host immune evasion is dominated by a SRS (SAG1-related sequence) family of proteins which includes the SAG1-like sequence branch and the SAG2-like sequence branch [5]. SRS proteins are expressed in a stage-specific manner. SAG1, SAG2A, SAG2B, SAG3, SRS1, SRS2, and SRS3 are mainly expressed on the tachyzoite surface [6]. Studies have indicated that SAG2 members participate in the process of parasite's invasion to the host, and their antibodies could block the further attachment of *T. gondii* on host cells [7, 8]. Previous studies have demonstrated that *T. gondii* parasites with a deletion of SAG2C, -2D, -2X, and -2Y gene cluster are less capable of maintaining a chronic infection in the brain [9]. It revealed that SAG2CDXY are important for persistence of cysts in the brain and these antigens might protect bradyzoites against an immune response. Contrary to SAG2A and SAG2B, which are expressed in tachyzoites, SAG2C, -2D, -2X, and -2Y appeared to be expressed exclusively on the surface of bradyzoites [9, 10]. However, among 160 members of the SRS family, only three proteins' structures were reported. They are (i) the tachyzoite-expressed SAG1 [11], (ii) the bradyzoite-expressed BSR4 [12], and (iii) the sporoSAG [13]. The structure and function domains of SAG2C, -2D, -2X, and -2Y are still not very clear.

In this study, we sought to predict the structure and function domains of SAG2C, -2D, -2X, and -2Y by bioinformatics methods. The protein sequence alignments were performed by the T-Coffee method. Secondary structural and functional domains were predicted using the software PSIPRED v3.0 and SMART software. The 3D structure model of each protein was mapped using the I-TASSER server. The structural similarities of these proteins were summarized and possible functions of some key amino acids were predicted using the space confrontation by VMD and SWISS-spdbv. Furthermore, HLA-restricted epitopes of SAG2C, -2D, -2X, and -2Y proteins were predicted via algorithms.

## 2. Methods

### 2.1. Data Resources

The protein sequences were derived from ToxoDB 5.1 (http://toxodb.org/toxo/). *Toxoplasma gondii* has three common types: type I, *T. gondii* GT1 (TGGT1_chrX 7,429,598); type II, *T. gondii* ME49 (TGME49_chrX 7,419,075); type III, *T. gondii* VEG (TGVEG_chrX 7,553,721). The original resources are listed in Table 1.

### 2.2. Modular Architecture Identification

Multiple sequence alignment tool, T-Coffee (http://www.tcoffee.org/) [14, 15], was used to obtain the alignment analysis among SAG2C, SAG2D, SAG2X, and SAG2Y. The secondary structures were constructed using the software PSIPREDv3.0 (http://bioinf.cs.ucl.ac.uk/psipred/) [16, 17]. Simple modular architecture research identification and annotation of signaling domain sequences were analyzed via a web-based tool, SMART (http://smart.embl-heidelberg.de/) [18].

The 3D models of proteins were constructed by I-TASSER, a protein structure server on the website http://zhanglab.ccmb.med.umich.edu/I-TASSER/, which is considered to predict protein 3D structures that have more than 100 amino acids [19–21]. VMD is a molecular visualization software for displaying, animating, and analyzing large biomolecular systems using 3D graphics and built-in scripts (http://www.ks.uiuc.edu/Research/vmd/). VMD was used to read standard Protein Data Bank (PDB) files and display the contained structure [22–25]. Swiss-Pdb Viewer (http://www.expasy.org/spdbv/) is an application that provides a user friendly interface allowing analyses of several proteins at the same time. The proteins can be superimposed in order to obtain structural alignments and compare their active domains. We deduced amino acid mutations, H bonds, angles, and distances between atoms from the intuitive graphic and menu interface. 3D protein molecular fitness analysis was performed for SAG2C, -2D and SAG2X, -2Y [22, 23].

### 2.3. Conserved HLA-Restricted Epitopes Prediction

Consensus methods including ANN, SMM, and CombLib-Sidney in immune epitope database IEDB (http://www.immuneepitope.org/) were used to predict HLA-restricted epitopes [26–28]. We used this tool to determine each peptide sequence's ability to bind to the specific HLA class I molecule.

## 3. Results and Discussion

### 3.1. Amino Acid Sequence Alignment Analysis

**Figure 1:**
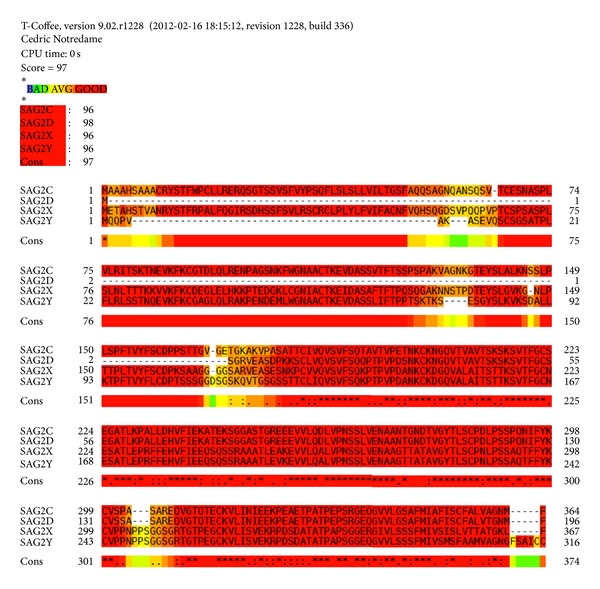
Alignment analysis for SAG2C, -2D, -2X, -2Y proteins. T-Coffee: multiple sequence alignment tools were used to obtain the alignment analysis result for SAG2C, -2D, -2X, and -2Y. Color bar indicated the identity, from bad identity to good identity.

SAG2C, SAG2D, SAG2X, and SAG2Y are positioned next to each other on chromosome X. The molecular masses of SAG2C, -2D, -2X, and -2Y are 32–38 kDa, 18–20 kDa 31–34 kDa, and 28–30 kDa, respectively [9]. Multiple sequence alignment for SAG2C, -2D, -2X, and -2Y shows that the four proteins sequences have 97% similarity (Figure 1). In Particular, SAG2C (184 to 364) has a 98% sequence identity to SAG2D (14 to 196) and SAG2X (184 to 367) has a 99% sequence identity to SAG2Y (128 to 300). The protein sequence alignment analysis indicated that SAG2C, -2D, -2X, and -2Y have high homologous sequences. However, when including SAG2A and SAG2B in the alignment analysis, the consensus dropped to 73%, even though the consensus between SAG2A and SAG2B has very good score 84%. It indicates that a great difference exists among SAG2A, -2B and SAG2C, -2D, -2X, -2Y.

### 3.2. 2-D Structure Alignment for SAG2C, -2D, -2X, and -2Y Proteins

**Figure 2:**
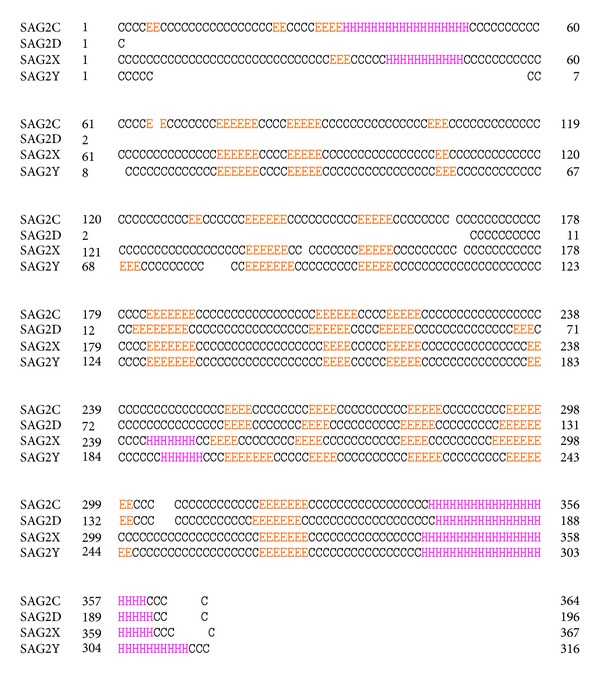
The 2D Structures of SAG2C, -2D, -2X, and -2Y proteins. PSIPREDv3.0 was used to predict the secondary structure for SAG2C, -2D, -2X, and -2Y proteins (C stands for coil, H stands for *α*-helix, and E stands for *β*-strand).

PSIPRED v. 3.0 was used to predict the secondary structures of SAG2C, -2D, -2X, and -2Y proteins.  Figure 2 showed that SAG2C protein has two *α*-helixes, 19 *β*-strands, and 20 coils; SAG2D protein has one *α*-helix, 9 *β*-strands, and 10 coils; SAG2X protein has 3 *α*-helixes, 14 *β*-strands, and 18 coils; SAG2Y protein has two *α*-helixes, 15 *β*-strands, and 18 coils. Obviously, there was a long *α*-helix on the C-terminal of all the proteins. SAG2D protein has similar secondary structure elements as SAG2C protein resides from 169 to 364. SAG2X and SAG2Y also have quite similar secondary structures except for a little discrepancy: SAG2X have one more helix than SAG2Y and one strand less than SAG2Y.

**Figure 3:**
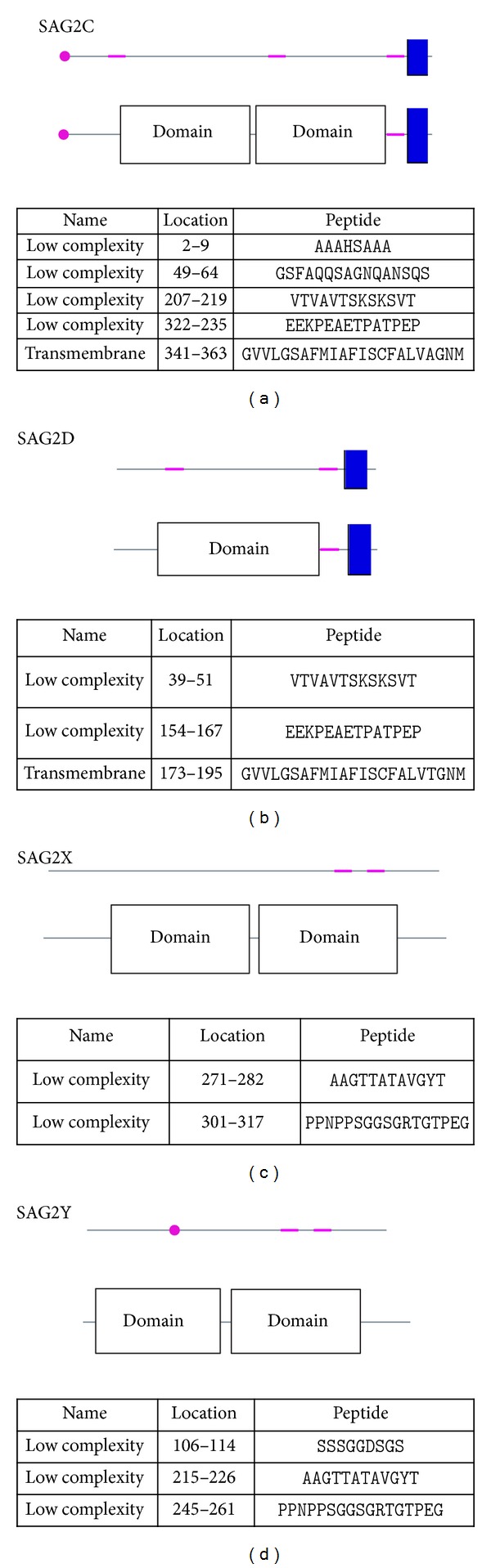
Prediction for protein domain. A web-based tool—SMARTM was used to figure out the domains of these proteins: transmembrane segments predicted by the TMHMM2 program (segments in blue color), segments of low compositional complexity determined by the SEG program (segments in purple color), signal peptides determined by the SignalP program (segments in red color), and domain (segments in gray color).

Furthermore, we used SMART to identify domains of these proteins (Figure 3). SAG2C, SAG2X, and SAG2Y all have two domains, while SAG2D only has one domain. SAG2D has an insertion of an adenosine, causing a frame shift and a premature stop codon, presumably leading to a truncated protein. SAG2C and SAG2D have transmembrane segments, while no transmembrane segments were identified on SAG2X and SAG2Y. From Figure 3, we could see that these proteins have no signal peptides, indicating that they are mature proteins. Members of the SAG2 family also differ in terms of open reading frame size, with the smaller SAG2D protein consisting of only one SAG domain, whereas SAG2C, SAG2X, and SAG2Y contain two SAG domains interrupted by a single intron. This indicates that SAG2C, SAG2X, and SAG2Y proteins have similar structure domains except SAG2D protein, which only has one domain.

### 3.3. Construction of 3D Model for SAG2C, -2D, -2X, and -2Y Proteins

3D model of SAG2C, -2D, -2X, and -2Y proteins were constructed by I-TASSER server. Five models were set up for each protein by Dr. Zhang's lab [19]. We selected the model with highest confidence C-score, which estimates the quality of predicted models by I-TASSER. It was calculated based on the significance of threading template alignments and the convergence parameters of the structure assembly simulations [20]. C-score is typically in the range of [−5, 2], and model with a C-score above 2 suggested a high confidence.

Low temperature replicas (decoys) generated during the simulation were clustered by SPICKER and top five cluster centroids were selected to generate full atomic protein models. The cluster density was defined as the number of structure decoys at each unit of space in the SPICKER cluster. A higher cluster density meant that the structure occurs more often in the simulation trajectory and therefore a better quality model.  Table 2 showed the parameters for construction D model of each protein. 

**Figure 4:**
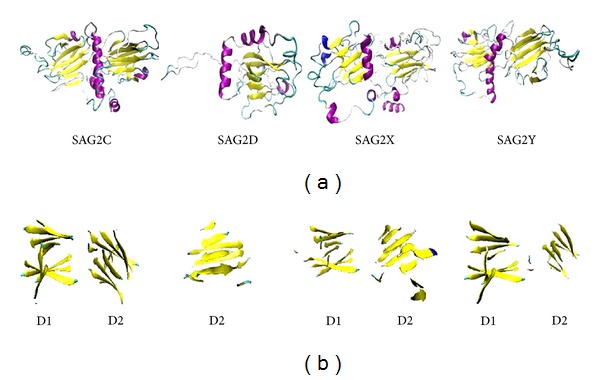
The 3D models of SAG2C, -2D, -2X, and -2Y. The sequences of proteins were sent to Dr. Zhang's lab from the website http://zhanglab.ccmb.med.umich.edu/I-TASSER/. The 3D models with the highest score for each protein were selected. The models were viewed by VMD software, color method was secondary structure (yellow: *β*-strands, purple: *α*-helix, gray: coil), and draw method was new cartoon. The domain of each model was shown out in sheet form.

The best model of each protein was selected and viewed via VMD program (Figure 4). SAG2C, -2X, and -2Y have obvious two domains, D1 and D2, which are formed by two *β*-strands separated by one *α*-helix; SAG2D has one domain which is formed by one *β*-strand separated by one *α*-helix. The *β*-strands rotate to form a sheet tube that is a common character of these proteins. Furthermore, the binding sites of residues in the model were predicted and showed in Table 3. 

Previous analysis of SAG2C, -2D, -2X, and -2Y structures revealed that the five on three *β* sandwich fold of SAG2 was most similar to the *T. gondii* bradyzoite-expressed BSR4 with TM-scores of 0.583, 0.661, 0.672, and 0.670, respectively (Table 4). BSR4 is a prototypical bradyzoite surface antigen encoded in a cluster of SRS genes on chromosome IV, including the closely related paralogs SRS6 and SRS9 [8, 9]. Sequence alignment shows that SAG2C, -2D, -2X, and -2Y share 71% sequence identity with the tachyzoite-expressed BSR4. This observation is consistent with the prediction that stage-specific structural features might play an important role in the process of infection, dissemination, and pathogenesis in *T. gondii*. In BSR4, two strands are organized in an antiparallel fashion, followed by another strand on the lower face of the *β* sandwich. The dimeric structure of SAG1 showed a *β* sandwich, two parallel outside strands with an opposite one in between [29]. The overall topology of the five on three *β* sandwich D2 domain is conserved between SAG2C, -2D, -2X, -2Y and BSR4. A detailed comparison of SAG2C, -2D, -2X, -2Y and BSR4 reveals a similarity in topology of the D1 and D2 domain consistent with the lower Z-score from the Dali search.

**Figure 5:**
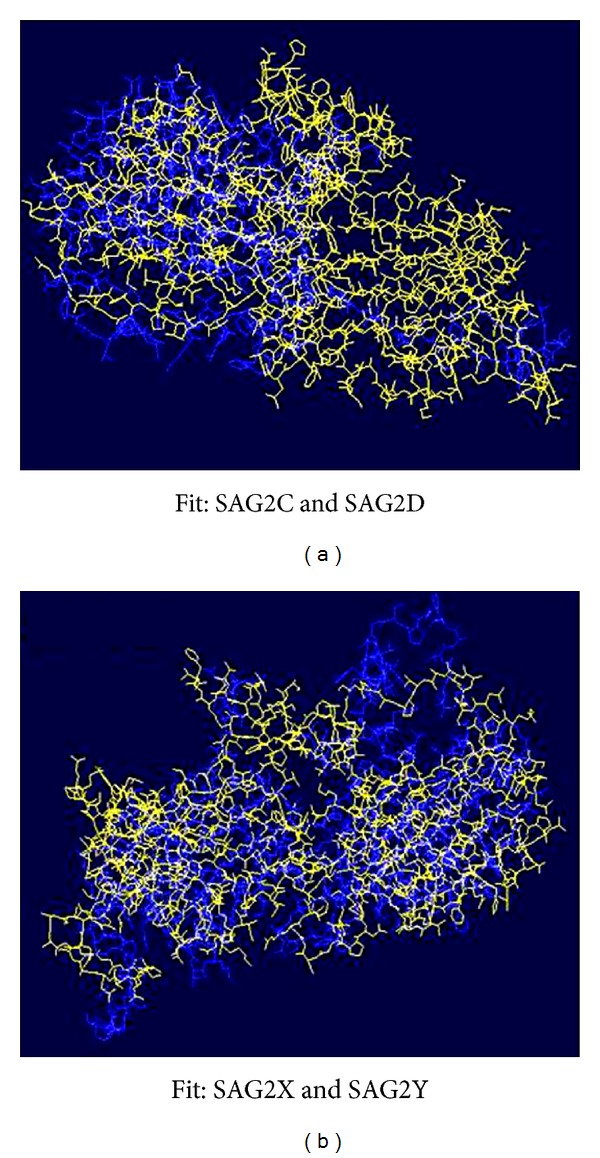
Fit analyses of SAG2C, -2D, -2X, and -2Y. Swiss-Pdb Viewer was used to show the fitness. SAG2C and SAG2X are in yellow color; SAG2D and SAG2Y are in blue color.

By comparison, the next most similar structure is SproSAG (surface antigen glycoprotein) with a substantially reduced TM-score [30, 31]. SporoSAG is a dominant surface coat protein expressed on the surface of sporozoites. SporoSAG crystallized as a monomer and displayed unique features of the SRS *β* sandwich fold compared to SAG1 and BSR4 [9]. Intriguingly, the structural diversity is localized to the upper sheets of the *β* sandwich fold and may have important implications for multimerization and host cell ligand recognition. By fit analysis, SAG2D fits well on the C-terminal of the protein SAG2C. SAG2X and SAG2Y fit pretty well from C-terminal to N-terminal (Figure 5).

### 3.4. Conserved HLA-Restricted CD8^+^ T Cells Epitope Prediction

Epitope prediction algorithm consensus was used to predict peptides that could stimulate human to induce effective and protective immune response against *T. gondii*. We want to see if they have similar epitopes scattered on the surface of their protein. The epitopes from SAG2C, -2D, -2X, and -2Y were predicted using the software from IEDB (http://www.immuneepitope.org/) which could identify novel HLA-class I restricted CD8^+^ T cell epitopes derived from *T. gondii*. 16 peptides were selected based on a high HLA allele binding score (percentile rank < 3). 

From Table 5, we can see three HLA-A*0201-restricted peptides: VVLGSAFMI, FMIAFISCF, AFISCFALV; four HLA-A*1101-restricted peptides: QVTVAVTSK, SSPQNIFYK, QVGTQTECK, KVLINIEEK; and two HLA-B*0702-restricted peptides: LPSSPQNIF, KPEAETPAT shared by SAG2C and SAG2D. From Table 6, we can see two HLA-A*0201-restricted peptides: ALVPNSSLV, VLSSSFMIV; three HLA-A*1101-restricted peptides: ALAITSTTK, SSAQTFFYK, KVLISVEKR; and two HLA-B*0702-restricted peptides: LPSSAQTFF, RPDSDATAT shared by SAG2X and SAG2Y.

**Figure 6:**
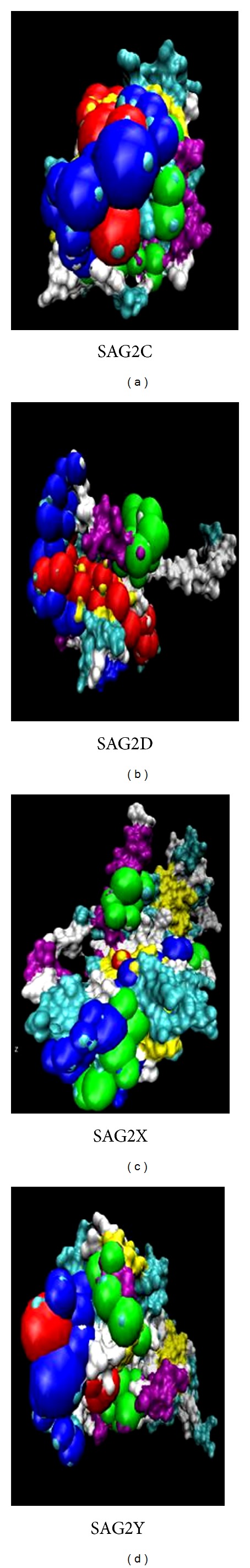
Identifying HLA-restricted epitopes on the surface of 3D models. The predicted HLA-restricted epitopes sequences shown in Tables 2 and 3 were marked out on the surface of 3D models of SAG2C, -2D, -2X, and -2Y. The 3D structures of proteins were shown using Surf method. Red color balls stand for epitopes restricted by HLA-A*1101, green color balls stand for epitopes restricted by HLA-A*0201, and blue color balls stand for epitopes restricted by HLA-B*0702.

More interestingly, when we marked the HLA-restricted epitopes on the alignment sequences of the proteins, we found that the epitopes restricted by the same type of HLA allele are located at the same domains of the proteins (Figure 6). Our results indicated that the epitopes from SAG2C, -2D, -2X, and -2Y can be recognized by the proper MHC-I molecular and present on the cell surface to induce immune response in the host CD8^+^ T cells which might be helpful on vaccine study and diagnosis for this parasitic disease. Some identified peptides from these proteins have been proven to be recognized by PBMC cells from proper HLA-restricted *T. gondii* seropositive individuals and significantly induced IFN-*γ* production in T cells from immunized mice [32, 33] and therefore confirmed our predictions.

## 4. Conclusions

In this study, we have conducted a detailed bioinformatic and structural characterization analysis of the bradyzoite proteins SAG2C, -2D, -2X, and -2Y. The characterization of SAG2C, -2D, -2X, and -2Y provided structural view of the *T. gondii* SRS family members at chronic bradyzoite stage. Our bioinformatic analysis clearly showed that SAG2C, -2D, -2X, and -2Y are homologous protein members of the SAG2 subfamily. Consistently, our structural analysis demonstrated that SAG2C, -2D, -2X, and -2Y are similar to two other bradyzoite SAG2 members, BSR4 and SPOROSAG, rather than tachyzoite SAG1. This result indicated that SAG2 family has conserved structure at bradyzoite stage but a great difference from SAG1 at tachyzoite stage. Furthermore, the predicted conserved peptides and HLA-restricted epitopes shed interesting light on vaccine study and diagnosis for this parasitic disease.

## Figures and Tables

**Table 1:** The original resources for SAG2C, D, X, and Y.

Protein	Size^a^	Coding gene location in types I, II, and III parasites^b^	Derivation^c^
SAG2C	365 aa	TGGT1_chrX: 7,358,316–7,360,225(−);	
		TGME49_chrX: 7353954–7,365860 (−);	SRS49D
		TGVEG_chrX: 7,440,957–7,442,866(−)	
SAG2D	196 aa	TGGT1_chrX: 7,352,615–7,353,874(−);	
		TGME49_chrX: 7,353,328–7,354,587(−);	SRS49C
		TGVEG_chrX: 7,435,249–7,436,508 (−)	
SAG2X	367 aa	TGGT1_chrX: 7,352,615–7,353,874(−);	
		TGME49_chrX: 7,353,328–7,354,587(−);	SRS49B
		TGVEG_chrX: 7,435,249–7,436,508 (−)	
SAG2Y	316 aa	TGGT1_chrX: 7,358,316–7,360,225(−);	
		TGME49_chrX: 7,355,817–7,356,572 (−);	SRS49A
		TGVEG_chrX: 7,440,957–7,442,866(−)	

^a^Size is the amino acid number that the protein has.

^b^Coding gene is the location of the gene that coded the protein.

^c^SRS domain-containing protein number.

**Table 2:** Parameters for predicted best 3-D model.

Name	C-score^a^	TM-score^b^	RMSD^b^	No. of decoys^c^	Cluster density^d^
SAG2C	−2.18	0.46 ± 0.15	11.8 ± 4.5	460	0.0285
SAG2D	−1.45	0.54 ± 0.15	8.5 ± 4.5	4662	0.0842
SAG2X	−2.22	0.45 ± 0.15	11.9 ± 4.4	455	0.0280
SAG2Y	−1.59	0.52 ± 0.15	9.9 ± 4.6	2877	0.0525

^a^C-score is a confidence score for estimating the quality of predicted models by I-TASSER. C-score is typically in the range of [−5, 2], where a C-score of higher value signifies a model with a high confidence and vice versa.

^b^TM-score and RMSD are known standards for measuring structural similarity between two structures which are usually used to measure the accuracy of structure modeling when the native structure is known.

^c^Number of decoys represents the number of structural decoys that are used in generating each model.

^d^Cluster density represents the density of cluster.

**Table 3:** Prediction binding site residues in the model.

Protein model	C-score^LB^ ^a^	TM-score^b^	RMSD^c^	IDEN^d^	Cov.^e^	BS-score^f^	Lig. name	Predicted binding site residues in the model
SAG2C	0.09	0.350	7.07	0.031	0.604	0.84	MAL	222, 223, 224, 225, 226, 227, 273, 279
SAG2D	0.11	0.435	4.90	0.043	0.668	0.75	ANP	96, 153, 155
SAG2X	0.06	0.388	5.94	0.053	0.597	0.79	NA	261, 292, 326
SAG2Y	0.07	0.412	6.36	0.057	0.687	0.77	FES	144, 145, 146, 147, 149, 150, 151, 153, 243

^a^C-score^LB^ is the confidence score of predicted binding site. C-score^LB^ values range between [0-1], where a higher score indicates a more reliable ligand-binding site prediction.

^b^TM-score is a measure of global structural similarity between query and template protein.

^c^RMSD is the RMSD between residues that are structurally aligned by TM-align.

^d^IDEN is the percentage sequence identity in the structurally aligned region.

^e^Cov. represents the coverage of global structural alignment and is equal to the number of structurally aligned residues divided by length of the query protein.

^f^BS-score is a measure of local similarity (sequence and structure) between template binding site and predicted binding site in the query structure.

**Table 4:** Similarity between SAG2C, -2D, -2X, -2Y and top identified structural analogs BSR4 protein.

Protein model	TM-score^a^	RMSD^b^	IDEN^c^	Cov.^d^
SAG2A	0.583	1.20	0.244	0.596
SAG2B	0.661	2.42	0.167	0.755
SAG2C	0.673	1.68	0.179	0.698
SAG2D	0.670	2.85	0.194	0.763

^a^TM-score of the structural alignment between the query structure and known structures in the PDB library.

^b^RMSD is the RMSD between residues that are structurally aligned by TM-align.

^c^IDEN is the percentage sequence identity in the structurally aligned region.

^d^Cov. represents the coverage of the alignment by TM-align and is equal to the number of structurally aligned residues divided by length of the query protein.

**Table 5:** Predicted HLA restricted CD8^+^ T cell epitopes for SAG2C, -2D of *T. gondii. *

Allele	Sequence	Pecentile rank	Method used	Location	
				SAG2C	SAG2D
HLA-A*0201	VVLGSAFMI	3.4	Consensus (*ANN, SMM, CombLib Sidenev * 2008)	342–350	174–182
HLA-A*0201	FMIAFISCF	1.4	Consensus (*ANN, SMM, CombLib Sidenev 2008*)	348–356	180–188
HLA-A*0201	AFISCFALV	2.8	Consensus (*ANN, SMM*)	351–359	183–191
HLA-A*1101	QVTVAVTSK	1.45	Consensus (*ANN, SMM*)	206–214	38–46
HLA-A*1101	SSPQNIFYK	0.2	Consensus (*ANN, SMM*)	290–298	122–130
HLA-A*1101	QVGTQTECK	2.35	Consensus (*ANN, SMM*)	308–316	140–148
HLA-A*1101	KVLINIEEK	1	Consensus (*ANN, SMM*)	316–324	148–156
HLA-B*0702	LPSSPQNIF	1.1	Consensus (*ANN, SMM, CombLib Sidenev 2008*)	288–296	120–128
HLA-B*0702	KPEAETPAT	2.6	Consensus (*ANN, SMM, CombLib Sidenev 2008*)	324–332	156–164

**Table 6:** Predicted HLA-restricted CD8^+^ T cell epitopes for SAG2X, -2Y of *T. gondii. *

Allele	Sequence	Pecentile rank	Method used	Location	
				SAG2C	SAG2D
HLA-A*0201	ALVPNSSLV	1.6	Consensus (*ANN, SMM, CombLib Sidenev 2008*)	260–268	204–212
HLA-A*0201	VLSSSFMIV	1.2	Consensus (*ANN, SMM, CombLib Sidenev* 2008)	346–354	290–298
HLA-A*1101	ALAITSTTK	1.6	Consensus (ANN, SMM)	208–216	152–160
HLA-A*1101	SSAQTFFYK	0.1	Consensus (*ANN, SMM*)	289–297	234–242
HLA-A*1101	KVLISVEKR	2.75	Consensus (*ANN, SMM*)	311–319	263–271
HLA-B*0702	LPSSAQTFF	3	Consensus (*ANN, SMM, CombLib Sidenev 2008*)	285–293	232–240
HLA-B*0702	RPDSDATAT	2.2	Consensus (*ANN, SMM, CombLib Sidenev 2008*)	314–322	271–279
